# Lead Ions Encapsulated in Liposomes and Their Effect on *Staphylococcus aureus*

**DOI:** 10.3390/ijerph10126687

**Published:** 2013-12-02

**Authors:** Renata Kensova, Iva Blazkova, Marie Konecna, Pavel Kopel, Dagmar Chudobova, Ondrej Zitka, Marketa Vaculovicova, David Hynek, Vojtech Adam, Miroslava Beklova, Rene Kizek

**Affiliations:** 1 Department of Chemistry and Biochemistry, Faculty of Agronomy, Mendel University in Brno, Zemedelska 1, Brno CZ-613 00, Czech Republic; E-Mails: R.Kensova@seznam.cz (R.K.); iva.blazkova@seznam.cz (I.B.); mariekon@centrum.cz (M.K.); paulko@centrum.cz (P.K.); dagmar.chudobova@centrum.cz (D.C.); ZitkaO@seznam.cz (O.Z.); Marketa.Ryvolova@seznam.cz (M.V.); d.hynek@email.cz (D.H.); vojtech.adam@mendelu.cz (V.A.); 2 Central European Institute of Technology, Brno University of Technology, Technicka 3058/10, Brno CZ-616 00, Czech Republic; E-Mail: beklovam@vfu.cz; 3 Department of Ecology and Diseases of Game, Fish and Bees, Faculty of Veterinary Hygiene and Ecology, University of Veterinary and Pharmaceutical Sciences, Palackeho 1-3, Brno CZ-612 42, Czech Republic

**Keywords:** lead, liposome, toxicity, differential pulse voltammetry, cyclic voltammetry, atomic absorption spectrometry, IC_50_, *Staphylococcus aureus*

## Abstract

The aim of the study was the preparation of a liposome complex with encapsulated lead ions, which were electrochemically detected. In particular, experiments were focused on the potential of using an electrochemical method for the determination of free and liposome-encapsulated lead and determination of the encapsulation efficiency preventing the lead toxicity. Primarily, encapsulation of lead ions in liposomes and confirmation of successful encapsulation by electrochemical methods was done. Further, the reduction effect of the liposome matrix on the detected electrochemical signal was monitored. Besides encapsulation itself, comparison of toxicity of free lead ions and lead ions encapsulated in liposome was tested. The calculated IC_50_ values for evaluating the lead cytotoxicity showed significant differences between the lead enclosed in liposomes (28 µM) and free lead ions (237 µM). From the cytotoxicity studies on the bacterial strain of *S. aureus* it was observed that the free lead ions are less toxic in comparison with lead encapsulated in liposomes. Liposomes appear to be a suitable carrier of various substances through the inner cavity. Due to the liposome structure the lead enclosed in the liposome is more easily accepted into the cell structure and the toxicity of the enclosed lead is higher in comparison to free lead ions.

## 1. Introduction

Lead is a heavy metal, toxic at extremely low doses and it has the acute and chronic effects on human health. It shows multi-system tissue toxicity, causing neurological, cardiovascular, renal, gastrointestinal, hematologic, reproductive, genotoxic and carcinogenic effects [1]. Thanks to the negative characteristics of lead, it is necessary to monitor its amount in the body [2,3,4,5,6]. Moreover, some complexing agents could enhance or lower toxicity of this metal, thus the effects of such complexes should be considered. Liposomes are among such complexing agents. They are spherical nanoshells composed of lipid bilayers that enclose an aqueous phase. They are easily produced and stable in solution for a long time with no significant changes in size or structure [7]. Due to their small and controllable size (from tens to thousands of nanometers) and the presence of internal cavities, liposomes are the most investigated organic nanoparticles [8]. The internal cavity can be used for transporting enzymes, proteins, DNA, drugs and other substances [9,10,11]. Highly biocompatible liposomes can be modified by antibodies, protein receptors or radioactive substances [7,12,13]. Voltammetric methods can be used for characterization of the electroactive compounds in liposome cavity [14,15]. This study is focused on the application of electrochemical method for the determination of free and liposome-encapsulated lead and determination of the encapsulation efficiency preventing lead toxicity.

## 2. Results and Discussion

**Figure 1:**
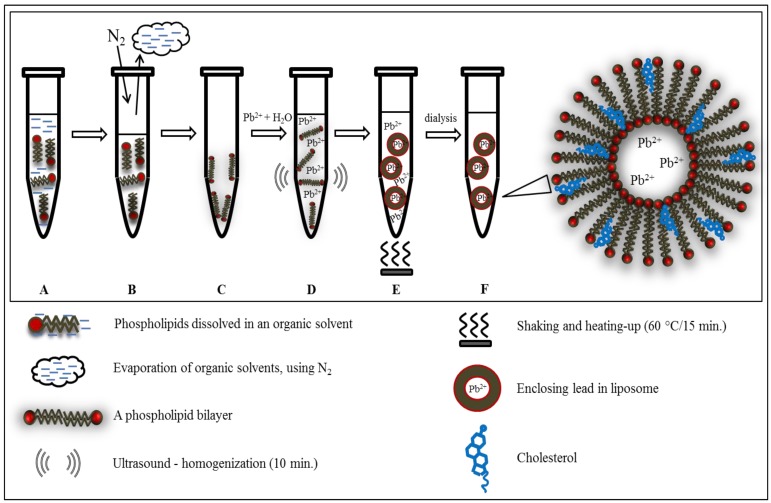
Scheme of lead encapsulation into the liposome structure. (**A**) Phospholipids were dissolved in chloroform. (**B**) After evaporation of the solvent (**C**) A lipid film was obtained. (**D**) Lead solution was added to the phospholipids bilayer. Samples were homogenized for 10 min using ultrasound. (**E**) The homogenized mixtures were heated and shaken for 15 min at 60 °C at Thermomixer Comfort (Eppendorf, Hamburg, Germany). (**F**) Non-captured lead ions were removed from the solution by dialysis.

The first and basic step of the experiment was encapsulation of lead ions into the liposome cavity. Lead was chosen as a model substance because metals often form part of an electrochemical label. Examples of such systems are so called quantum dots, which are nanocrystals most often composed of metal sulphides or tellurides [16]. Detailed sample preparation is described in the Experimental section and in Figure 1. The creation of this nanostructure was tested using electrochemical methods and atomic absorption spectrometry (AAS). Electrochemical techniques are one of the best methods for detecting metals due to their low cost, high sensitivity and potential portability [17,18,19,20,21]. Atomic absorption spectrometry was applied as a method for total lead determination. The verification of the encapsulation process was done by comparison of the amounts of lead determined by electrochemical methods and AAS. Because of the fact, that the electrochemical method is able to determine the lead on the surface of liposomes (and free in the solution) and on the contrary, AAS determines the total amount of lead (inside and outside of liposomes),the obtained difference should be thus related to the encapsulated lead amount.

### 2.1. Cyclic Voltammetry of Lead—Liposome Complex

**Figure 2:**
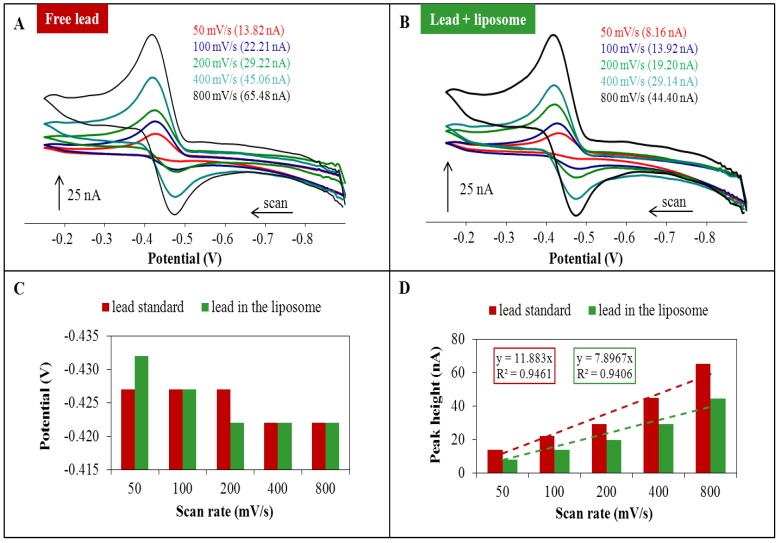
(**A**) Cyclic voltammograms of lead standards with concentration of 20 µM. (**B**) Cyclic voltammograms of lead in the liposome with concentration of 20 µM. (**C**) Changing the position of the lead standard peak and peak of lead encapsulated in the liposome depending on the applied scan rate. (**D**) Linear dependence for the peak heights of the lead standard signals and lead encapsulated in liposome signals on the applied scan rate. Lead was determined by CV method using different scan rates (50–800 mV/s). 0.2 M acetate buffer (pH = 5) was used as the supporting electrolyte. The characteristic peak for lead was at a potential of −0.4 V.

A comparison of the signal changes of lead encapsulated in liposomes and a lead standard measured by CV is shown in Figure 2. With the increasing scan rate the lead signal increased (Figure 2A,B). It is clear from the results shown in Figure 2C that the change of the lead peak potential is not expressly influenced by scan rate. Lead standard signals and signals of lead in the liposomes are compared in Figure 2D. Lead concentrations in both samples were 20 µM. However, the signals of lead in liposomes were lower than the signals of samples containing lead standard only. Therefore, one may suggest that the liposome matrix reduces the detection of lead in the samples. Analytical outputs in Figure 2D are the slopes of linear dependence for lead standard y = 11.883x, R^2^ = 0.95 and lead in liposomes y = 7.897x, R^2^ = 0.94, which indicates that the slope for lead in liposomes is lower.

### 2.2. Lead Encapsulation in Liposome

**Figure 3:**
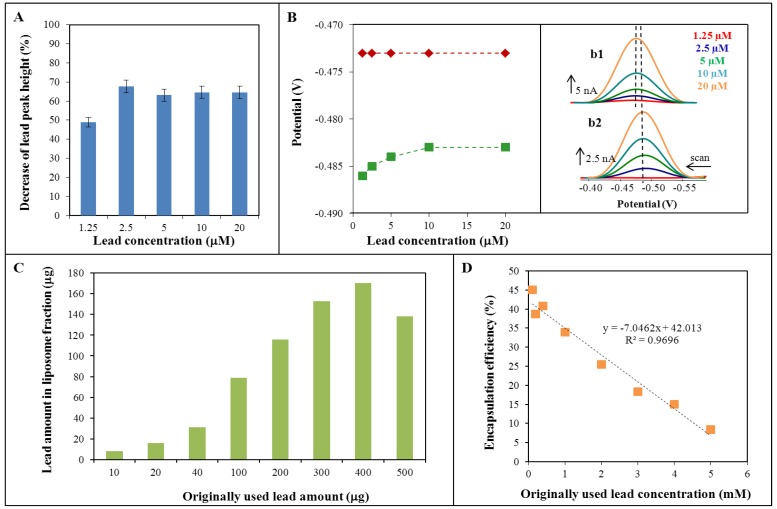
(**A**) Percentage decrease of lead signal by DPV method caused by the presence of liposome. (**B**) Change of the potential location of lead peaks determined with/without the presence of liposome. Inset b1: real voltammograms of free lead ions. Inset b2: real voltammograms of lead ions with liposome. (**C**) Lead amount in liposome fraction (created by dialysis) dependent on the originally used amount of lead (for the preparation of lead-liposome complexes). (**D**) The dependence of encapsulation efficiency on the originally used lead concentration.

Differential pulse voltammetry (DPV) was the second electrochemical method which was used for lead determination. The influence of the presence of liposomes (in measured lead solutions) on the obtained electrochemical signal was monitored (Figure 3A). It was seen that the lead peak height depended on the various lead concentrations (within the range from 1.25 to 20 µM) and compared to the signal intensity of free lead in the same concentration range. The resulting decrease in signal intensity is shown in Figure 3A. The average value of signal decrease is 61.8%. The obtained signals showed the change in peak potential too (Figure 3B). Peaks detected for free lead (lead nitrate standard solution) were located at −0.473 V, while the peaks related to the lead-liposome mixture were located approximately at −0.485 V. While the free lead determination peak potential was independent on the lead concentration, in the case of liposomes the peak potential was slightly dependent on the lead concentration (Figure 3B) - the growing lead concentration caused the potential shift to a more positive value.

For the investigation of liposome encapsulation efficiency the amount of lead in the liposomal fraction (created by dialysis) was monitored. Because lead is low molecular weight substance it is necessary to suggest that some part will be located on the surface of liposome structures. Therefore the amount of lead in the liposomal fraction was monitored by AAS to obtain maximum lead amount that could possibly be immobilized in this fraction (according to the original liposome concentration, see Experimental). The dependence of the amount of lead in the liposomal fraction related to the originally used amount of lead is presented in Figure 3C. It is obvious that the maximum amount of immobilized lead was about 170 µg. Because the difference between originally used lead amounts 300 and 400 g led to similar results, it should be assumed that the maximum amount of immobilized lead lies in the range from 152 to 170 µg.

The calculation of liposome encapsulation efficiency was based on the difference in the lead determination by AAS and DPV, as was mentioned above. The encapsulation efficiency was calculated in the concentration range of lead from 0.1 to 5 mM. The percentage of encapsulation efficiency related to the lead concentration is shown in Figure 3D. The calculated percentage was determined as the difference in the concentrations (obtained from above mentioned methods) and related to the originally used lead concentration. From the presented results it is obvious that the measured values of lead enclosed in liposomes were distinctly lower in comparison with the originally used lead concentrations. The efficiency of encapsulation decreased with increasing original lead concentration, as follows: y = −7.046x + 42.013, R^2^ = 0.97. This means that application of a lower original lead concentration led to a better result and, according to the previous paragraph, the application of an original lead concentration in the range from 0.1 to 2 mM is suitable. Since it was found that the application of lower original lead concentrations resulted in higher efficiency of the capture of lead ions in the liposomes, for sequent experiments µM concentrations of lead were chosen.

**Figure 4:**
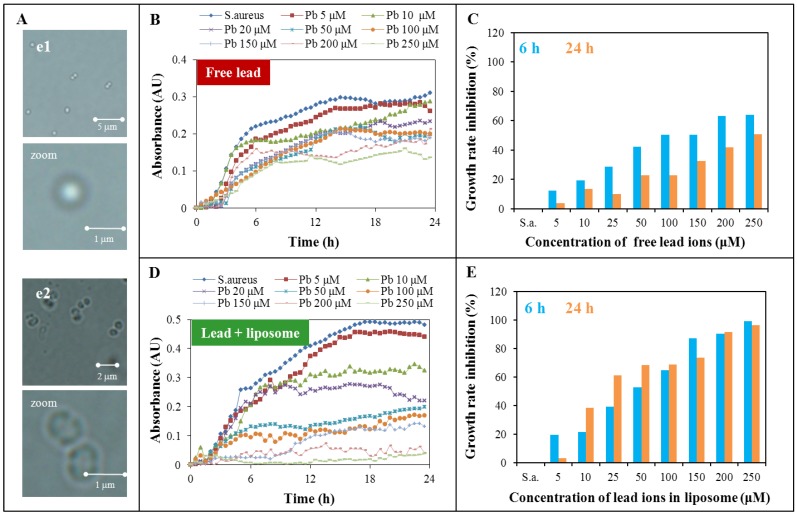
Spectrophotometric analysis of growth of the *S. aureus* in the presence of different concentrations of lead ions. (**A**) Microscopy images of *S. aureus* cells: (**e1**) Micrographs of control *S. aureus* cells (and enlarge image). (**e2**) Micrographs of cells after application of lead ions (250μM) (and enlarge image). (**B**) Growth curves of *S. aureus* treated with different concentrations of lead ions (5, 10, 20, 50, 100, 150, 200 and 250μM) without liposome. (**C**) Spectrophotometric analysis of the growth of *S. aureus* bacterial culture treated with lead ions concentrations of 5, 10, 20, 50, 100, 150, 200 and 250μM after 6 and 24 h. (**D**) Growth curves of *S. aureus* treated with different concentrations of lead ions (5, 10, 20, 50, 100, 150, 200 and 250μM) enclosed in liposome. (**E**) Spectrophotometric analysis of the growth of *S. aureus* bacterial culture treated with lead ions concentrations of 5, 10, 20, 50, 100, 150, 200 and 250μM after 6 and 24 h.

### 2.3. Toxicity Determination of Free Lead Ions and Lead Encapsulated in the Liposome

It is common knowledge that heavy metal ions cause inhibition of microorganism (bacterial) growth, therefore they are often incorporated into different kinds of materials to ensure antimicrobial activity [22,23]. The growth curves method serves as a tool to evaluate antimicrobial activity, as the effect of heavy metal ions on bacterial strains such as *Staphylococcus aureus* in this experiment [24,25,26]. It is possible to calculate the inhibition concentration value (IC_50_) from these curves via statistical methods [27]. Eight different concentrations of lead ions (5, 10, 20, 50, 100, 150, 200 and 250 μM) free (Figure 4A,B) or encapsulated into liposome (Figure 4C,D) were added to the bacterial strain *S. aureus* in this experiment.

The application of heavy metal ions to *S. aureus* cells led to significant morphological changes, observed in the cells in terms of cell shapes and the cell wall thicknesses. The difference between control and *S. aureus* cells after lead ion application is presented in Figure 4A. The cell wall was considerably thickened and the presence of so called cross walls (septal midline) was observed. These inner transverse walls are formed due to the developing resistance. Similar phenomenon is commonly observed in the case of methicillin-resistant strains of *S. aureus* commonly called MRSA strains [28,29,30].

The growth curves of *S. aureus* treated with both lead variants (free lead ions and lead ions encapsulated in liposomes) are presented in Figure 4B,D. The growth was the most rapid in the first six hours of measurement; therefore the growth rate of each variant was compared to *S. aureus* control. The same evaluation was done after 24 h. This time period was chosen to represent the long-term effect of lead on *S. aureus*. The growth curves show that the lead ions both enclosed in liposomes and free had antimicrobial activity and this growth inhibition was more significant with the increasing lead ions concentration. The inhibition effect of individual lead concentrations and forms (free and encapsulated) are presented in Figure 4C,E. The presented growth rate inhibition was calculated as the percentage of difference between the growth rate of the *S. aureus* control and the individually treated variants related to the *S. aureus* control. Figure 4C presents results related to the system with free lead ions for 6 and 24 h. Presented results show that the long-term effect of free ions is lower about 16% on average compared to the short-term effect. The maximum inhibition effect was achieved for the concentration of 250 µM for both time periods; after 6 h there was a 64% and for 24 h a 51% inhibition effect. More rapid increase of the inhibition effect was observed with the variants of lead in liposomes (Figure 4E) compared to the variants without liposomes. Application of liposome encapsulation caused a reduction of the differences between the short and long-term effects. This difference was reduced to 8% on average. Free lead ion application produced a maximal inhibition in 6 h of 64% The same inhibition efficiency was achieved using lead encapsulated in liposomes at a concentration 100 µM, which is 2.5 times lower. Similarly, application of 29 µM encapsulated lead concentration had the same effect in a 24 h time period as 250 µM in the case of free lead ions. From these results it is obvious that the effect of liposomes is more evident in the long-term evaluation. It is necessary to add the information about the maximum inhibition effect for encapsulated lead which occurs at 250 µM and was about 99% for 6 h and 96% for 24 h.

The statistically calculated IC_50_ values for evaluating the lead cytotoxicity are summarized in Table 1. Presented values show significant differences between the lead enclosed in liposomes and free lead. The various IC_50_ values confirm one important. After 24 h the difference between the IC_50_ values for free and encapsulated lead is so different that this suggestion could be considered as confirmed. Similar results are presented in another related works [31,32,33,34,35].

**Table 1:** Calculated values of IC_50_ (as micro molar concentration of lead) for various times of *S. aureus* incubation.

Hours	Free lead	Encapsulated lead
6	10.3	43.4
12	57.9	31.9
18	315.6	25.5
24	236.9	28.0

Another important obvious trend is visible in the free ion application where the inhibition effect fades with time. An opposite trend is visible in the case of liposome encapsulation. Here the inhibition effect grows with a maximum at 18 h (but the difference at 24 h is small). These two opposite trends confirm, in fact, the basic fact about lead encapsulation because various ways of lead transport into *S. aureus* cell had to be done. The shorter way of free lead ions and the longer way for the liposome transport into cells. Another important thing is more stable time effect of encapsulated lead compared to free ions. A more stable time effect is necessary to understand in concentration range, as there is a small concentration difference between the various IC_50_ values during the whole time period (24 h).

## 3. Experimental Section

### 3.1. Chemicals and Materials

Cholesterol, 1,2-dioleoyl-sn-glycero-3-phospho-rac-(1-glycerol) sodium salt, chloroform, Pb(NO_3_)_2_ and water (for liposome preparation) of ACS purity quality were purchased from Sigma-Aldrich (St. Louis, MO, USA). Hydrogenated phosphatidylcholine from soybeans was a gift from Lipoid GMBH (Ludwigshafen, Germany). To pipette volumes down to micro- and nanolitres, pipettes were purchased from Eppendorf Research (Eppendorf, Hamburg, Germany). The water (for bacterial cultivation) was prepared using Aqual 25 reverse osmosis equipment (Brno, Czech Republic). The water was further purified by using a MiliQ Direct QUV apparatus equipped with the UV lamp (Aqua Osmotic, Tisnov, Czech Republic). The resistance was 18 MΩ. The pH was measured using a WTW inoLab pH meter (Weilheim, Germany).

### 3.2. Preparation of Liposome

Liposomes were prepared by modification of a published method [36]. Cholesterol (100 mg), 1,2-dioleoyl-sn-glycero-3-phospho-rac-(1-glycerol) sodium salt (100 mg) and phosphatidylcholine (100 mg) were dissolved in chloroform (4.5 mL). A lipid film was obtained by rotary evaporation of solvent and residual chloroform was blown off with nitrogen.

### 3.3. Preparation of Liposome Filled with Lead

Solutions containing 0, 0.5, 1, 2.5 and 5 mg of lead (in the form of lead nitrate) were added to liposomes (20 mg). Samples were homogenized with an Ultra-Turrax T8 (IKA Werke GMBH, Staufen, Germany) for 10 min. The homogenized mixtures were then heated and shaken for 15 min at 60 °C at Thermomixer Comfort (Eppendorf). The samples were then washed several times with water on Amicon 3k (Millipore, Billerica, MA, USA). Final volume of samples was 1 mL.

### 3.4. Electrochemical Determination

#### 3.4.1. Differential Pulse Voltammetry (DPV)

Measurement of lead by DPV were performed with a 746 VA Trace analyser connected to a 695 Autosampler (Metrohm, Zofingen, Switzerland), using a standard cell with three electrodes. For data processing the VA Database 2.2 software provided by Metrohm CH was employed. The parameters of the measurement were as follows: initial potential of −1.2 V, end potential 0.15 V, deposition potential −1.2 V, deposition 480 s, deoxygenating with argon 60 s. Dosage was 25 µL of the sample to 1,975 µL of acetate buffer pH = 5.

#### 3.4.2. Cyclic Voltammetry (CV)

Determination of lead by CV were performed with a 797 VA Computrace instrument connected to a 813 Compact Autosampler (Metrohm), using a standard cell with three electrodes. For data processing the 797 VA Computrace software by Metrohm CH was employed. The parameters of the measurement were as follows: initial potential of −1.2 V, first vertex potential 0.15 V, second vertex potential −1.2 V, sweep rate 50–800 mV/s, deposition potential −1.2 V, deposition 480 s, deoxygenation with argon 60 s. Before the measurement 25 µL of the sample was added to the 1,975 µL of 0.2 M acetate buffer pH = 5.

### 3.5. Atomic Absorption Spectrometry (AAS)

Determination of lead was carried out on an 240FS Agilent Technologies atomic absorption spectrometer (Agilent Technologies, Santa Clara, CA, USA) with flame atomization. The lead hollow cathode lamp (Agilent) was operated at the current of 10 mA. Lead was measured at the wavelength of 217.0 nm with spectral bandwidth of 1.0 nm. The mixture of air and acetylene was used for flame atomization. Deuterium background correction was used and the signal was measured in integration mode for 2 s.

### 3.6. Toxicity Determination of Free Lead Ions and Lead Encapsulated in the Liposome

#### 3.6.1. Cultivation of *Staphylococcus aureus*

*Staphylococcus aureus* (NCTC 8511) was obtained from the Czech Collection of Microorganisms, Faculty of Science, Masaryk University, Brno, Czech Republic. Strains were stored as a spore suspension in 20% (*v*/*v*) glycerol at −20 °C. Prior to use in this study, the strains were thawed and the glycerol was removed by washing with distilled water. The composition of cultivation medium was as follows: 10 g tryptone, 5 g yeast extract and 5 g NaCl/L MilliQ water (Duchefa Biochemie, Haarlem, The Netherlands).The pH of the cultivation medium was adjusted at 7.4 before sterilization. Sterilization of media was carried out at 121°C for 30 min in a Tuttnauer 2450EL sterilizer (Tuttauer, Breda, The Netherlands). The prepared cultivation media were inoculated with bacterial culture in 25 mL Erlenmeyer flasks. After inoculation, bacterial cultures were cultivated for 24 h on a shaker at 600 rpm and 37 °C. Bacterial culture cultivated under these conditions [37,38] was diluted by cultivation medium to OD_600_ = 0.1 and used in the following experiments.

#### 3.6.2. Growth Curves

The procedure for the evaluation of the antimicrobial effect of the tested compounds involved measuring the absorbance using a Multiskan EX apparatus (Thermo Fisher Scientific, Waltham, MA, USA) and subsequent analysis in the form of growth curves [39,40]. The 24 h grown culture of *S. aureus* was diluted with LB medium (10 g tryptone, 5 g yeast extract, 5 g NaCl/L deionised water) using a Specord 210 spectrophotometer (Analytik Jena, Jena, Germany) at wavelength of 600 nm to absorbance 0.1. In the microplate *S. aureus* culture was mixed with various concentrations of lead in or without liposomes in different concentrations or *S. aureus* alone as a control for measurements. The concentrations of lead were 5, 10, 20, 50, 100, 150, 200 and 250 μM. Total volume in the microplate wells was 300 µL. Measurements were carried out at time 0, and every 30 min for 24 h at 37 °C at the wavelength of 620 nm. The values achieved were individually analysed in graphic form as growth curves for each group.

#### 3.6.3. Statistical Evaluation

The software STATISTICA (data analysis software system), version 10.0 (StatSoft, Tulsa, OK, USA) was used for data processing. Half-maximal concentrations (IC_50_) were calculated from logarithmic regression of sigmoideal dose-response curves. A general regression model was used to analyse differences between the combinations of compounds. To reveal differences between cell lines, Turkey’s *post hoc* test within homogenous groups was employed. Unless noted otherwise, *p* < 0.05 was considered significant.

#### 3.6.4. Cell Microscopy

An inverted system microscope Olympus IX 71 (Tokyo, Japan) was used for imaging the cells. The cells in cultivation medium were pipetted (5 μL) onto a microscope slide and covered by a cover slip. The sample was placed cover slip down and the immersion oil was used. The objective (PlanFLN; Mag. 100×; NA 1.3; F.N. 26.5) and the magnification lens 1.6× were used; the total magnification was 1,600×. The images were captured by an Olympus DP73 camera and processed by the Stream Basic 1.7 Software. The image resolution was 4,800 × 3,600 pixels. The parameters were as follows: exposure time: 32 ms and ISO 200.

## 4. Conclusions

Liposomes appear to be a suitable carrier of various substances due to their inner cavity. This ability makes liposomes among the most researched organic nanoparticles. The lead encapsulation into the liposome structure and the influence of the liposome matrix on the electrochemical detection of lead was confirmed. Lead encapsulated in the liposomes is more easily accessible to the cells of *S. aureus* and its toxicity in comparison with free lead ions is higher. In addition, we have demonstrated that electrochemical methods could be very suitable tools for the determination of electroactive compounds enclosed in liposomes.
